# Characterization and digital spatial deconvolution of the immune microenvironment of intraductal oncocytic papillary neoplasms (IOPN) of the pancreas

**DOI:** 10.1007/s00428-023-03543-4

**Published:** 2023-04-22

**Authors:** Antonio Pea, Gaetano Paolino, Filippo Martelli, Elena Bariani, Paola Piccoli, Elisabetta Sereni, Roberto Salvia, Rita T. Lawlor, Liang Cheng, David Chang, Aldo Scarpa, Claudio Luchini

**Affiliations:** 1 Department of Surgery, the Pancreas Institute, University and Hospital Trust of Verona, 37134 Verona, Italy; 2 The Institute of Cancer Sciences, University of Glasgow, G128QQ, Glasgow, UK; 3 Department of Diagnostics and Public Health, Section of Pathology, University of Verona, 37134 Verona, Italy; 4 ARC-Net Research Center, University and Hospital Trust of Verona, 37134 Verona, Italy; 5 Department of Pathology and Laboratory Medicine, Brown University Warren Alpert Medical School, Lifespan Academic Medical Center, and the Legorreta Cancer Center at Brown University, Providence, RI 02903 USA; 6 West of Scotland Pancreatic Unit, Glasgow Royal Infirmary, G40SF, Glasgow, UK

**Keywords:** Oncocytic, IPMN, IOPN, TME, Artificial intelligence, Digital pathology

## Abstract

Intraductal oncocytic papillary neoplasm (IOPN) of the pancreas is a distinct entity from intraductal papillary mucinous neoplasms (IPMNs) and is considered one of the precursor lesions of pancreatic cancer. Through immunohistochemistry (IHC) and an artificial intelligence (AI)-based approach, this study aims at characterizing its immune microenvironment. Whole-slide IHC was performed on a cohort of 15 IOPNs, 2 of which harboring an associated adenocarcinoma. The following markers were tested: CD3, CD4, CD8, CD20, CD68, CD163, PD-1, PD-L1, MLH1, PMS2, MSH2, and MSH6. The main findings can be summarized as follows: (i) CD8+ T lymphocytes were the predominant immune cells (*p* < 0.01); (ii) the vast majority of macrophages were concurrently CD68+ and CD163+; (iii) all tumors showed an activated PD-1/PD-L1 axis, but none had mismatch repair deficiency; (iv) AI-based analysis revealed the presence of 2 distinct regions in each case, namely, Re1, localized at the center of the tumor, and Re2, located at tumor periphery; (v) the infiltrating component of the 2 invasive IOPNs showed a smaller extent of Re1 and a reduced rate of CD4+ cells, as well as a larger extent of Re2 and increased rate of CD8+ cells. IOPNs are lesions enriched in immune cells, with a predominance of CD8+ T lymphocytes and class 2 macrophages. Differently from IPMN-oncogenesis, the progression towards invasive carcinoma is accompanied by an increased rate of CD8+ lymphocytes. This finding may suggest the presence of an active self-immune surveillance in invasive IOPNs, potentially explaining, at least in part, the excellent survival rate of IOPN patients.

**Supplementary Information:**

The online version contains supplementary material available at 10.1007/s00428-023-03543-4.

## Introduction

Intraductal oncocytic papillary neoplasm (IOPN) of the pancreas is an epithelial neoplasm characterized by the presence of complex branching papillae lined by oncocytic cells, growing within pancreatic ductal tree [1]. In the latest WHO classification, it is considered as a distinct subtype from the others intraductal neoplasms, including intraductal papillary mucinous neoplasms (IPMNs) [2]. Overall, IOPN accounts for 4–5% of all intraductal pancreatic neoplasms [2].

Histologically, IOPNs are composed of multiple layers of cuboid cells with abundant granular eosinophilic cytoplasm, large nuclei with round borders, and prominent eccentric nucleoli [3, 4]. From the immunohistochemical point of view, the most useful markers for supporting the diagnosis of IOPN are MUC5AC, above all in the differential diagnosis with intraductal tubulopapillary neoplasms, and MUC6, Hep Par-1, and CD117 for the differential diagnosis with IPMNs [2, 5]. On the molecular level, IOPNs are characterized by the absence of *KRAS* and *GNAS* activating mutations*,* which are typically found in pancreatic IPMNs and in pancreatic ductal adenocarcinomas (PDACs). Mutations in genes such as *ARHGAP26*, *ASXL1*, *EPHA8, and ERBB2* are more common [6], but fusions involving *PRKACA* and *PRKACB* genes have been recently described as the genetic hallmark of pancreatic and biliary IOPNs [7, 8].

IOPNs usually reach considerable size, and are associated with an invasive carcinoma in up to 30% of cases. When present, the invasive component is generally focal, and is usually composed of oncocytic cells arranged in a tubular-nested architecture [9–11]. Despite a relatively high prevalence of an associated infiltrating component, the prognosis of IOPNs is excellent after surgical resection, with a 5-year overall survival rate approaching 100%.

Interestingly, IOPNs are also typically associated with a marked infiltration by inflammatory cells, and the composition of the immunologic microenvironment has been described as a potential mediator of cancer invasion and metastasization [12–14]. Through immunohistochemistry assisted by computational approaches on digitalized slides, this study aims to provide a characterization of the composition and spatial distribution of the immune microenvironment of IOPNs. This effort may have implications on the understanding of the biological peculiarities of this type of lesions.

## Materials and methods

This study has been approved by the Verona Ethical Committee (project: EPAT-2020, number of approval: 2801/CESC, date of approval: 24-06-2020) and has been conducted in accordance with the Good Practice guidelines, the Declaration of Helsinki, and current laws, ethics, and regulations. All cases with a diagnosis of IOPN were retrieved from the ARC-Net biobank at Verona University Hospital. We selected only cases for which tumor slides and blocks were available.

One representative formalin-fixed paraffin-embedded (FFPE) tissue block was selected for each case for whole-section immunohistochemistry (IHC), which was performed as already described [15, 16]. Briefly, 4 μm, FFPE sections were immunostained with the following antibodies, according to the manufacturer’s instructions: CD3 (clone: LN10; dilution: 1:200; Bio-Optica, Italy), CD4 (clone: 4B12; dilution: 1:100; Novocastra, Germany), CD8 (clone: C8/144B; dilution: 1:200; Dako, USA), CD20 (clone: L26; dilution: 1:100; Novocastra. Germany), CD68 (clone: KP1; dilution: 1:400, Dako, USA), CD163 (clone: 10D6; dilution: 1:200; Novocastra, Germany), PD-1 (clone: NAT; dilution: 1:100; Abcam, UK), PD-L1 (clone: SP263, pre-diluted 0.05 M, Roche, Switzerland), MLH1 (clone: ES05; dilution: 1:30; Dako, USA), PMS2 (clone: MRQ-2; dilution: 1:150; Cell Marque, USA), MSH2 (clone: FE11; dilution: 1:30; Dako, USA), and MSH6 (clone: EP49; dilution: 1:100; Dako, USA). Heat-induced antigen retrieval was performed using a heated plate and 0.01 mol/l of citrate buffer, pH 8.9, for 15 min. Light nuclear counterstaining was performed with hematoxylin (5 min). All samples were processed using a sensitive peroxidase-based “Bond polymer Refine” detection system in an automated Bond instrument (Vision-Biosystem, Leica, Milan, Italy). Sections incubated without the primary antibody served as negative controls.

IHC slides were evaluated separately and in blind by two pathologists (G.P., C.L.). Inconsistencies were resolved by consensus at a multi-headed microscope. For CD3, CD4, CD8, CD20, CD68, CD163, and PD-1, IHC was considered positive when the cell membrane was stained. The evaluation of the expression of these biomarkers was performed using a semi-quantitative (0–5) scoring system, as reported elsewhere [17, 18]: 0 = negative (no positive cells), 1 = rare (1–10 positive cells per high power field — HPF, 40X), 2 = low (11–20 positive cells per HPF), 3 = moderate (21–30 positive cells per HPF), 4 = high (31–50 positive cells per HPF), and 5 = very high (>50 positive cells per HPF). PD-1 evaluation was based on the single most positive HPF, while the scores for CD3, CD8, CD4, CD20, CD68, and CD163 were obtained as a mean value of the five most positive HPFs, as already described [19].

For MLH1, PMS2, MSH2, and MSH6, IHC was considered positive (retained expression) when the cell nuclei was stained, as already described and per current guidelines [20]. The evaluation of the expression of these biomarkers was classified as positive (retained expression) or negative (expression loss). PD-L1 expression was evaluated as reported elsewhere [21–24], and using two specific scores: (i) tumor proportion score (TPS) which assesses the percentage of positive viable tumor cells, showing partial or complete membrane staining at any intensity, and (ii) combined positive score (CPS) which takes into account the number of tumor and non-neoplastic PD-L1 positive cells, compared with all viable tumor cells.

**Fig. 1:**
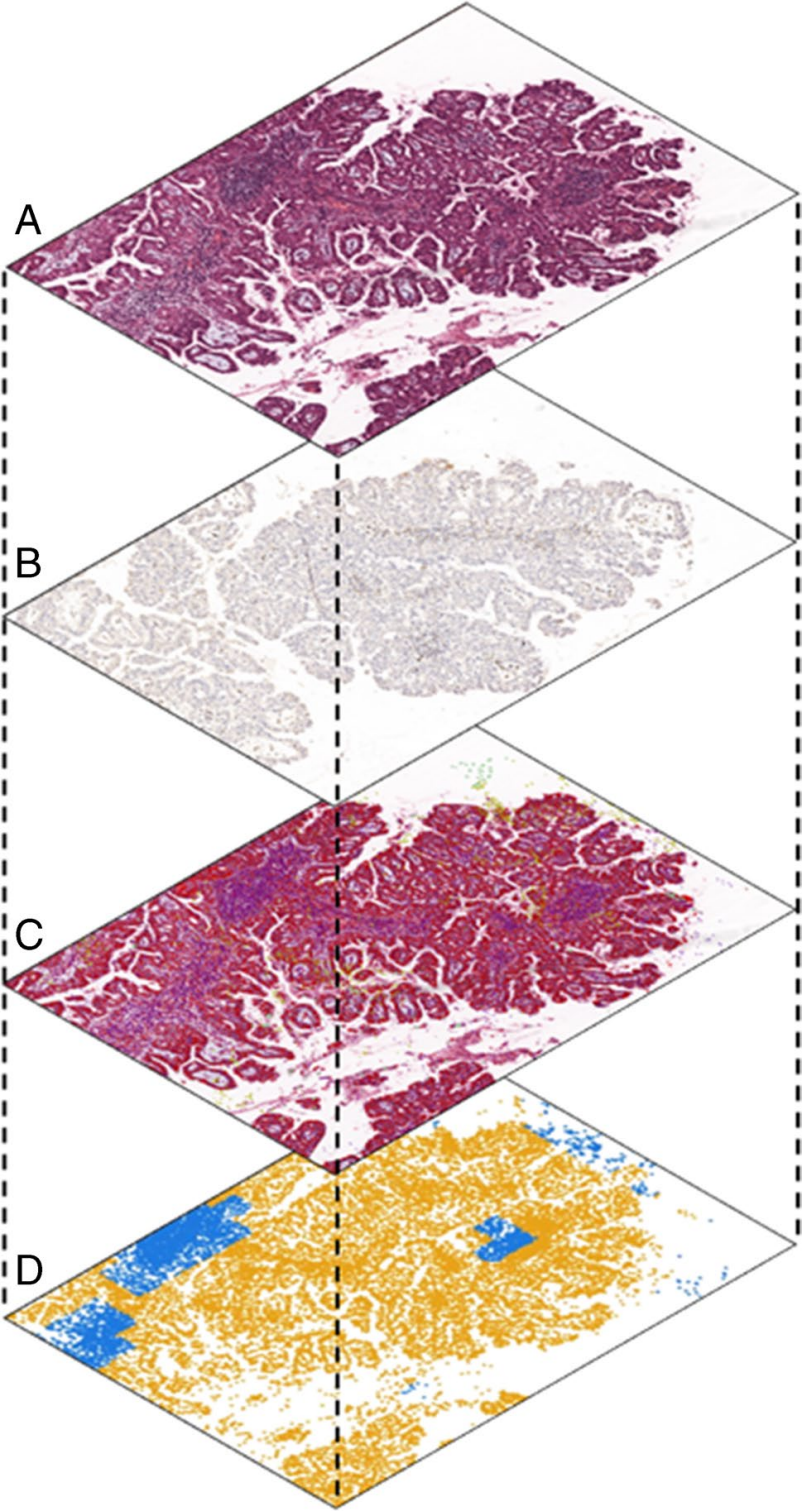
Workflow of imaging processing. A random tree forest classifier was generated to identify tumor immune and stromal cells on hematoxylin-eosin (H&E) images (**A**); on immunohistochemical (IHC) images, positive cells were automatically detected (**B**). Serial H&E and IHC sections were spatially aligned to virtually project the different immune phenotypes from each IHC section on the H&E image, generating a composite map including cancer cells and all immune subsets (**C**). On the composite map, spatial relationship among immune cells and cancer cells was computed through distance, density, and “neighborhood” analysis, generating a color-coded positional plot using Cytomap software (**D**)

The patterns of immunohistochemical expression of the lymphocytes markers CD4, CD8, and CD20 (CD4 and CD8 for T cells; CD20 for B-cells) have been also evaluated in terms of stain intensity and spatial disposition using artificial intelligence-based algorithms (Fig. 1). Hematoxylin-eosin (H&E) slides and the matched CD4, CD8, and CD20-stained slides were digitized using the APERIO platform (Leica Biosystems) at 20× of magnification. Each slide was then analyzed using the QuPath open source software platform (version 0.2.3) [25]. A region of interest (ROI) was annotated for each hematoxylin-eosin (H&E) stained slides including the whole tumor and excluding any surrounding normal pancreatic/duodenal tissue, and transferred on the corresponding IHC slide. Cell detection was performed using Stardist [26], a digital pathology toolbox that utilizes star-convex polygons to localize nuclei, for each H&E and IHC slide. A random tree forest classifier for each H&E slide was generated using cell features to classify cells into tumor, immune, and stromal cells. To maximize the accurateness of the algorithm, smoothed features at 25 and 50 μm radius were added and multiple rounds of cell classification review were performed. For each IHC slide, positive cells were detected and transferred to the H&E slide following slides alignment (Fig. 1).

First, all IOPNs without an associated adenocarcinoma were analyzed. Then, a specific analysis was performed for IOPNs with an associated adenocarcinoma, where the two components (i.e., intraductal vs. infiltrative) were analyzed separately. Spatial analysis was performed by exploring the organization of immune cells and their relationship among them and with tumor cells using Cytomap, as already described [27]. Briefly, cells were grouped into 200 μm raster scanned neighborhoods, which are cylindrical windows where the position of each neighborhood is evenly distributed across the tissue in a grid pattern.

Then, neighborhoods were clustered into regions based on their cellular composition using “Self-Organizing Map” (SOM) model [28], with the number of regions determined by the Davies-Bouldin criterion [29]. Cellular composition was defined as the number of cells specifically positive for a given IHC marker in each neighborhood, divided by the total number of cells in that neighborhood.

## Results

Overall, a cohort of 15 IOPN have been collected. Of them, 2 cases showed an associated invasive adenocarcinoma. The most important clinicopathological features, as well as the results of the immunohistochemical analysis, are summarized in Table 1.

**Table 1:** Summarizing table of the most important clinicopathological features and IHC results (numerical scores) of this case series

ID	Gender	Age (years)	T (cm)	Type IPMN	Site	pTNM	CD3	CD4	CD8	CD20	PD-1	PD-L1		CD68	CD163	MLH1	PMS2	MSH2	MSH6
												TPS	CPS						
1	M	63	2	Mx	H	pTis	5	4	5	2	2	5%	5	4	3	+	+	+	+
2	M	41	14	Mx	B	pTis	5	4	5	1	3	0%	0	4	3	+	+	+	+
3	M	80	4	Mx	H	pTis	5	4	5	2	5	0%	0	3	3	+	+	+	+
4	M	56	5	Mx	H	pTis	5	3	5	4	5	50%	55	3	3	+	+	+	+
5	F	65	3,5	MD	B	pTis	4	1	4	0	1	0%	0	2	2	+	+	+	+
6	M	80	5	Mx	H	pTis	5	4	4	1	3	0%	5	3	2	+	+	+	+
7	M	60	5	Mx	B	pTis	5	3	5	2	5	10%	12	4	3	+	+	+	+
8	M	46	6	Mx	T	pTis	5	3	4	2	4	20%	22	3	3	+	+	+	+
9	F	68	4	Mx	H	pTis	4	2	3	0	1	0%	0	4	4	+	+	+	+
10	F	68	2	Mx	B	pTis	5	3	3	4	3	5%	5	4	4	+	+	+	+
11	M	65	8	Mx	H	pTis	4	2	3	3	5	60%	65	4	3	+	+	+	+
12	M	63	5	MD	B-T	pTis	5	3	4	1	4	0%	0	3	3	+	+	+	+
13	M	48	5	Mx	H	pTis	5	3	4	3	5	2%	10	3	2	+	+	+	+
14	M	62	3 (1)	BD	H	T1cN0M0	4 (5)	3 (2)	4 (5)	1 (1)	2 (3)	0% (0%)	5 (20)	2 (5)	2 (5)	+	+	+	+
15	M	60	6 (3)	BD	B	T2N0M0	3 (5)	4 (2)	3 (5)	2 (2)	1 (4)	0 (5%)	0 (7)	4 (5)	3 (5)	+	+	+	+
Mean values:			**5.2 (2)**	**-**	**-**	**-**	**4.6 (5)**	**3.1 (2)**	**4.1 (5)**	**1.9 (1.5)**	**3.3 (3.5)**	**10.1% (2.5%)**	**12.3 (13.5)**	**3.3 (5)**	**2.9 (5)**	**+**	**+**	**+**	**+**

In the case of an infiltrating component, its values are indicated within brackets

Bold emphasis was used in the last line providing the summary of all results listed in the table

*Abbreviations*: *ID* identification-number, *T* tumor size, *IPMN* intraductal papillary mucinous neoplasm; Type IPMN: *MD* main duct, *BD* branch duct, *Mx* mixed; Site: *H* head, *B* body, *T* tail; pTNM: tumor size, nodal metastasis, distant metastasis (pathological assessment); *PD-1* programmed death-1, *PD-L1* programmed death-ligand 1, *TPS* tumor proportion score, *CPS* combined positive score, *NA* not applicable

The vast majority of patients (80%) were male, and the mean age of the cohort was 61.6 years old. The mean cyst size was 5.2 cm, whereas the mean size of the two IOPN-associated invasive adenocarcinoma was 2 cm. The most common site was the pancreatic head (53.3%), and mixed involvement of the ductal tree was the most frequent presentation (73.3%).

Concerning IHC results, the first evident findings regarded the spatial organization of lymphocytes. CD20-positive B lymphocytes were mainly localized at the periphery of the tumor, with absent or very limited presence inside the tumor area. At the same time, CD3-positive T lymphocytes presented the highest density within tumor area (consistent with the so-called TILs, tumor-infiltrating lymphocytes) and these were mainly located in papillary cores on among tumor cells. Considering both tumor area and its periphery, there were more T lymphocytes than B, with a statistically significant difference in terms of IHC scores (mean IHC score: 4.6 vs. 1.9, *p* < 0.001, Student’s *t*-test). Regarding the composition of T lymphocytes, CD8-positive cells were present in a larger number than CD4-positive cells, with a statistically significant difference (mean IHC score: 4.1 vs. 3.1, *p* = 0.001, Student’s *t*-test). Regarding the infiltrating component of both invasive IOPNs, CD4+ cells showed reduced IHC scores, whereas CD8+ cells showed higher scores.

Macrophages were mainly located within tumor area. CD163-positive cells, a distinctive subclass of macrophages, showed almost superimposable patterns and very similar scores to CD68-positive cells, indicating that the vast majority of macrophages were positive for both CD68 and CD163. The IHC scores of both markers were higher in the infiltrating components of both invasive IOPNs.

The IHC results regarding PD-1/PD-L1 axis highlighted a high prevalence of expression of such biomarkers in IOPNs. PD-1 was expressed in TILs, and in all cases with IHC scores ranging from 1 to 5 and with a mean value of 3.3. At the same time, PD-L1 showed a TPS of 0 in 8/15 cases (53.3%), with a mean TPS of 10.1%, and a CPS of 0 in 6/15 cases (40%), with a mean CPS of 12.3. The presence of 2 cases with discordant TPS vs. CPS, i.e., cases #6 and #14, TPS = 0 and CPS = 5 in both cases, indicated that PD-L1 was expressed in such cases only in inflammatory cells and not in tumor cells. One case showed the presence of positive cells in the infiltrative component only, but without reaching high values (TPS = 5% and CPS = 7)

All mismatch-repair proteins, namely, MLH1, PMS2, MSH2, and MSH6, displayed retained expression on tumor cells (positive nuclear staining), indicating that there was no mismatch repair deficiency/microsatellite instability.

**Fig. 2:**
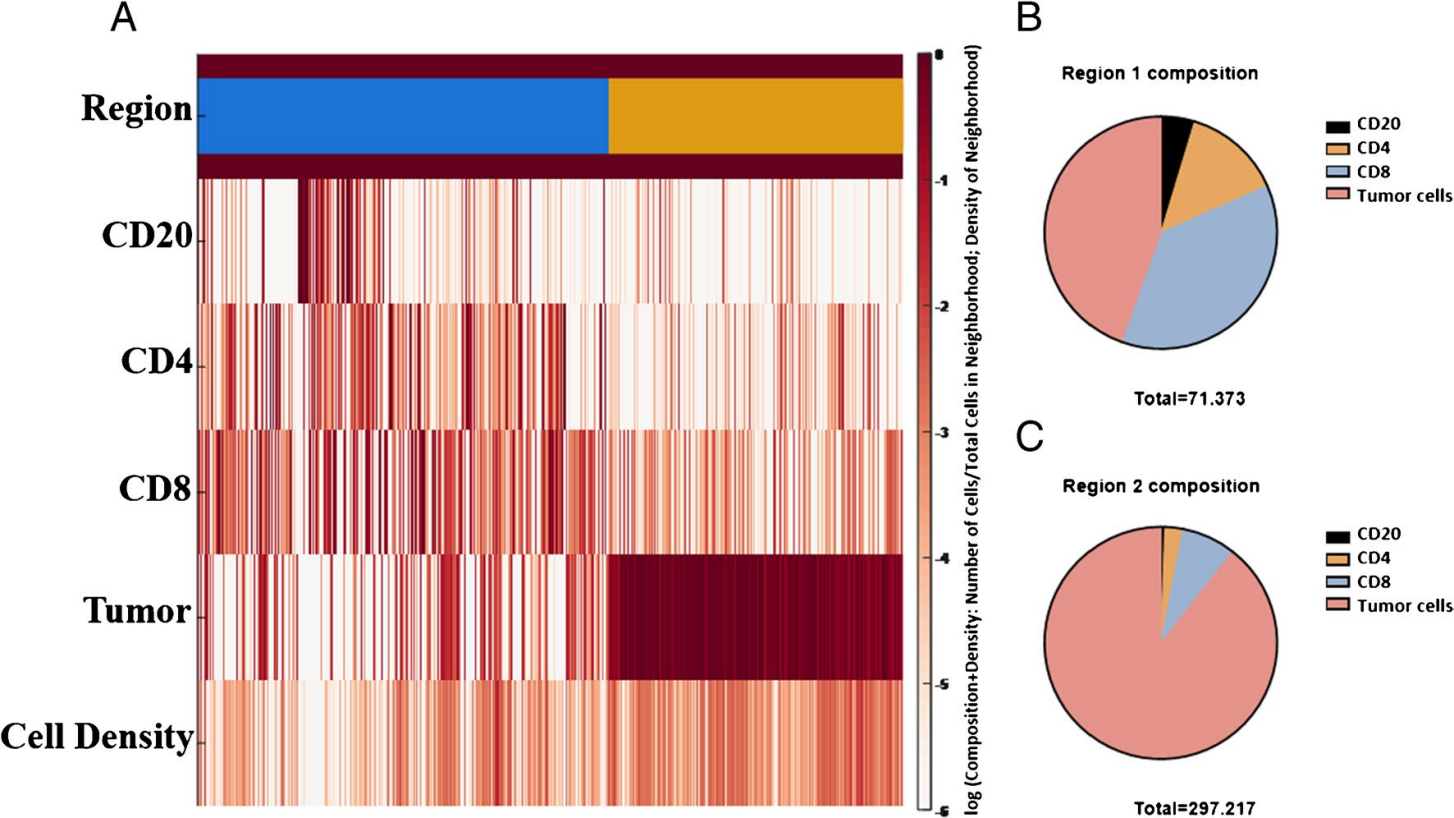
Heat-map and circle plots based on the different regions identified with Self-Organizing Map (SOM) analysis. Heat-map showing the cellular composition of the neighborhoods (percentage of each cell phenotype per neighborhood) after SOM clustering in region 1 (Re1; blue) and region 2 (Re2; yellow), and the relative prevalence of the regions combining all non-invasive samples (**A**). Circle plots demonstrating the median cellular composition per neighborhood (cells in each neighborhood/total number of cells in that neighborhood) for Re1 (**B**) and Re2 (**C**) are also presented. Re1 composition: CD20: 4.69%, CD4: 13.71%, CD8: 37.18%, tumor cells: 44.42%. Re2 composition: CD20: 0.54%, CD4: 2.54%, CD8: 7.50%, tumor cells: 89.43%

The digital pathology analysis with artificial intelligence-based algorithms was used to detect and count tumor cells and cellularity, and *B* vs. *T* cells (both CD4 and CD8 positive cells) in terms of IHC marker expression and spatial distribution. Regarding the 13 IOPNs without an associated invasive adenocarcinoma, SOM clustering analysis revealed the presence of 2 distinct regions in each case, which have been called region 1 (Re1), localized at the center of the tumor, and region 2 (Re2), located at tumor periphery, both characterized by a distinct composition in terms of neoplastic and immune cells (Fig. 2). Re1 displayed a decreased total cell density and a generally higher rate of immune cells, whereas Re2 showed higher density of tumor cells. The specific cell composition, case by case and based on Re1 vs. Re2 distinction, is reported in Supplementary Table 1.

**Fig. 3:**
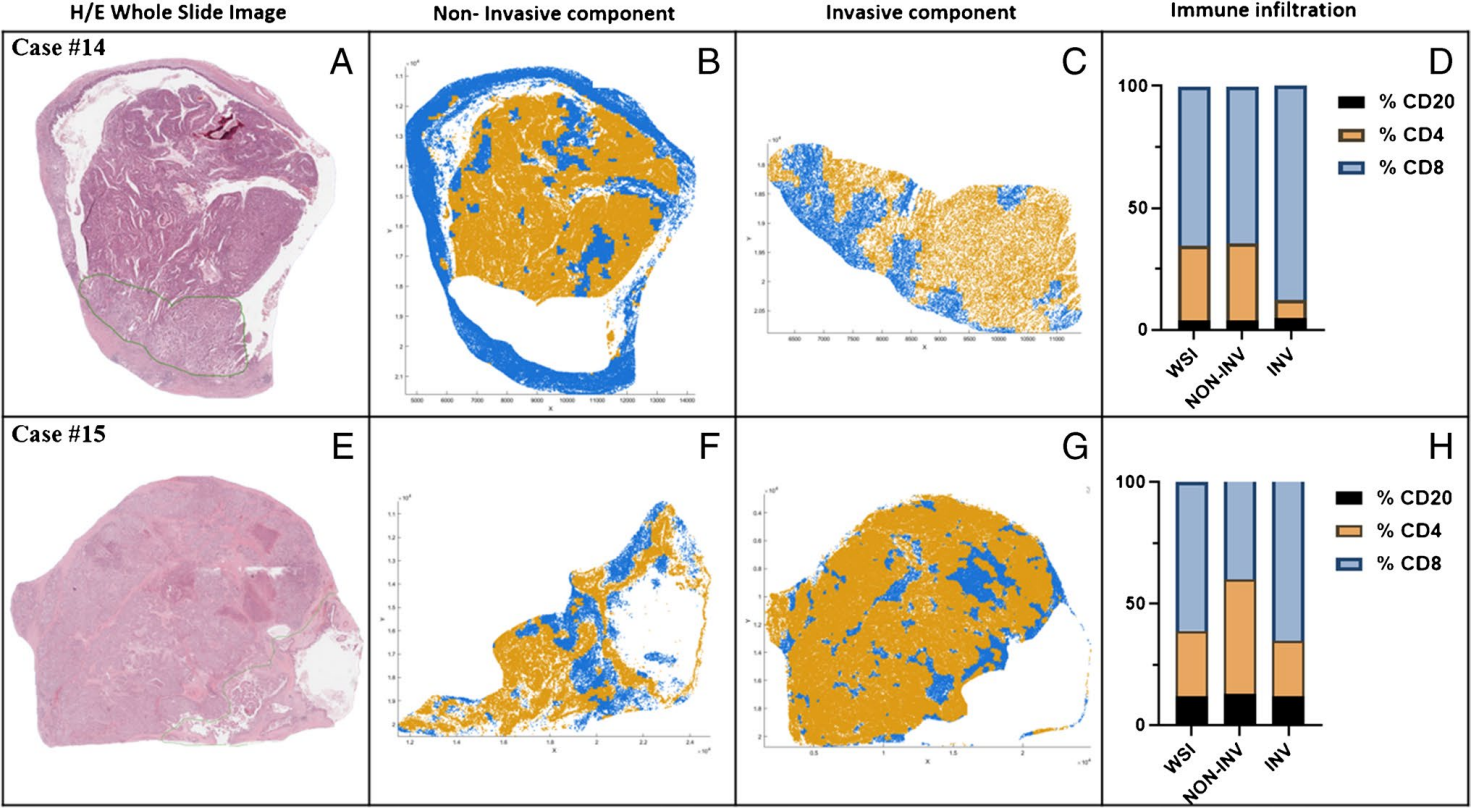
Specific analysis on the 2 invasive IOPNs (**A**–**H**). The region of interest (ROI) was manually placed onto each hematoxylin-eosin slide of the 2 cases of intraductal pancreatic neoplasm with associated invasive cancer, including epithelial and stromal tissue, and excluding non-neoplastic tissue with deep-learning approaches (**A**, **E**). Region color-coded positional plots of the neighborhoods of the non-invasive (**B**, **F**) and of the invasive components (**C**, **G**) showing cells included in region 1 (Re1; blue) and region 2 (Re2; yellow) are shown. Immune infiltration and cellularity (percentage of each phenotype on the total immune cells) in the entire slide (WSI) and in the non-invasive (NON-INV) and invasive (INV) components (**D**, **H**) are also presented

The specific analysis of the 2 IOPNs with an associated adenocarcinoma is summarized in Fig. 3. First, the same clustering algorithm used for the non-invasive IOPNs was also applied on these cases (on the entire slide, and separately on the invasive and non-invasive components). Sub-analysis demonstrated a lower extension of Re1 in the invasive cancer compared with the intraductal component (35% vs. 48% of the entire tumor in the first case, and 19% vs. 38% in the second case), and a larger extension of Re2 in the invasive cancer compared with the intraductal component (65% vs. 52% of the entire tumor in the first case, and 81% vs. 62% in the second case). We therefore investigated the immune infiltrate in the 2 separated components (intraductal vs. invasive), observing a reduced rate of CD4+ cells and an increased rate of CD8+ cells in the invasive component of both cases, with a statistical significant difference (*p* < 0.001 in both cases, Student’s *t*-test). Raw measurements and percentages of cell composition of the two components are listed in Supplementary Table 2.

## Discussion

In this study, we provided the characterization of the immune microenvironment of a cohort of 15 pancreatic IOPNs, 2 of which harboring an associated adenocarcinoma. IOPN tumor cells presented an activated PD-1/PD-L1 axis and a surrounding microenvironment characterized by a larger number of B cells than T cells (*p* < 0.001) and CD68+/CD163+ macrophages, mainly located within the tumor. Digital pathology modeling identified 2 distinct regions localized at the center and at periphery of the tumor and presenting a different immune composition.

A critical finding is that IOPNs are characterized by the presence of a remarkable number of inflammatory cells. In particular, T cells emerged as the most represented cell type not only in the periphery but also within the tumor mass, being present in both the vascular cores of papillae and among tumor cells. Among T cells, cytotoxic lymphocyte CD8+ were the most represented. Interestingly, the two cases associated with an invasive carcinoma showed an increased rate of CD8+ cells and a decreased rate of CD4+ cells in the infiltrative component. Along this line, a recent study reported the results of single-cell RNA sequencing of a cohort of IPMNs with low-grade and high-grade dysplasia, and with associated invasive carcinomas [13]. At the single-cell transcript level, this study showed different patterns of T cell infiltration, which were linked to the grade of IPMN dysplasia. Cases with low-grade dysplasia were enriched in CD4+ and CD8+ T cells. Intriguingly, instead of increasing, the populations of T lymphocytes decreased in a stepwise manner during progressive dysplasia, suggesting a decrease in the immune response or a tumor escape mechanism during neoplastic progression [13, 30]. The findings we observed in our IOPN cohort are quite different. First, IOPN are lesions composed, by definition, of neoplastic cells with high-grade dysplasia; thus, a stepwise progression from low-grade to high-grade areas cannot be assessed. Then, the associated carcinomas were characterized not only by a decreased infiltration of CD4+ T cells but also by an increased infiltration of CD8+ T cells. Such findings may indicate that IOPN-derived carcinomas do not acquire a complete tumor escape signature as observed in IPMNs, further corroborating the importance of differentiating IOPNs from IPMNs. Moreover, the rich immune microenvironment of IOPNs and the increase of CD8+ T cells during progression may explain, at least in part, the surprising excellent prognosis even in those cases harboring an invasive component. Indeed, these findings may support the persistence of immune surveillance in IOPNs during the acquisition of infiltrative potential. Not surprisingly, the PD-1/PD-L1 axis also appeared activated in our cohort, where all cases showed the presence of positive cells for PD-1, PD-L1, or both, and with increased scores in the invasive components.

Of note, digital pathology-based analysis revealed the presence of two distinct regions, namely Re1, at the center of the tumor, and Re2, at tumor periphery. Interestingly, the invasive components showed a lower extension of Re1 as well as a larger extension of Re2. This change in cellular spatial distribution and tumor architecture, as revealed by SOM clustering analysis, is accompanied by inflammatory cell perturbation, with a decrease of CD4+ cells and an increase in CD8+ cells. This observation suggests that not only tumor cells but also lymphocytes might play an active role in the acquisition of infiltrating capability. The presence of cytotoxic CD8+ T cells also in the invasive components of IOPN is a unique feature in the microenvironment of PDAC precursors, including IPMNs and pancreatic intraepithelial neoplasia (PanIN) [30–33]. Less likely the combination of high PD-L1 expression associated with higher CD8+ T cells in IOPN is associated with an “immune exhaustion” phenotype as observed in a PDAC subtype with poorer clinical outcomes. The different outcomes may be related to the differences in the number and types of presented antigens and relative immune responses by infiltrating cells in IOPN and PDACs. These observations may call for specific considerations in terms of treatment opportunities. Moreover, this type of lesion may serve as a model for studying anti-tumor immune strategies in the early phases of pancreatic carcinogenesis.

Along this line, another interesting topic may be represented by the analysis of macrophages. Tumor-associated macrophages (TAM) indeed represent a critical component of the tumor immune microenvironment, and are characterized by the expression of CD68 [34–36]. Two classes of TAM have been identified. The first is the so-called TAM1, which exhibits a pro-inflammatory phenotype and is thought to inhibit tumor development and extension; the second is TAM2, which expresses the marker CD163 and supports tumor growth and progression [34–36]. Although no data regarding CD163 expression in IOPN exist in the literature, this marker has already been indicated as a poor prognostic moderator in different cancer types including PDAC [37]. Given the almost superimposable staining pattern of CD68 and CD163 in our cohort, we showed that IOPN are very rich in TAM2/CD163+ macrophages. Of note, the IHC score of CD163 was higher in the infiltrative components of both invasive IOPNs, indicating a potential active role of TAM2 in the acquisition of infiltrating capabilities. Overall IOPN showed a peculiar immune infiltrate that presents important differences in term of cell components with those observed in conventional pancreatic cancer. These differences may be related to the relatively small infiltrative component in IOPN and with an immune system that may respond differently to early-stage cancer cells compared to more advanced cancer cells.

Our study does have some limitations. First, we investigated a relatively small sample size. However, IOPNs are quite a rare entity and of difficult pathological assessment, and this cohort size is in line with other studies of this tumor type in the literature. Furthermore, we have not investigated other immunohistochemical markers potentially useful to further characterize the immune cells in IOPNs microenvironment. At the same time, we focused our attention on the most distinctive biomarkers for defining the most important subclasses of tumor-associated immune cells. Finally, while all considerations in this study regarding potentially applicable models for studying immune modulation in pancreatic cancer are interesting, they are more explorative and speculative in nature than immediately feasible.

In conclusion, in this study, we provided the characterization of the immune microenvironment of a cohort of 15 IOPNs, 2 of which with an associated adenocarcinoma. We showed that this type of lesion is enriched in immune cells, with a predominance of CD8+ T lymphocytes and class 2 macrophages. The invasive component showed a reduced rate of CD4+ cells, but an increased rate of CD8+ lymphocytes and macrophages. The peculiarities observed in IOPNs in terms of immune cell populations and distribution further confirm their classification as a distinct entity among pancreatic intraductal lesions. Furthermore, the increase in CD8+ cells in the invasive components may suggest the presence of an active self-immune surveillance in these cases, potentially and partially explaining the excellent survival rate of patients with IOPN and associated invasive cancer. These lesions could also be tested as a model for investigating immunomodulation strategies in pancreatic cancer.

## Supplementary information

ESM 1: **Supplementary Table 1**. Summarizing table of cell composition for each region (cells in each neighborhood / total number of cells in that neighborhood) in the 13 non-invasive IOPNs. **Supplementary Table 2**. Summarizing table of the number of cells and relative percentage in the two IOPNs with associated invasive adenocarcinoma.

## Data Availability

All data and materials as well as software application or custom code support their published claims and comply with field standards.
