# Supplementary material for: Characterization and digital spatial deconvolution of the immune microenvironment of intraductal oncocytic papillary neoplasms (IOPN) of the pancreas
Source: Virchows Arch. 2023 Apr 22;483(2):157–65. doi: 10.1007/s00428-023-03543-4 (PMC10412653; doi:10.1007/s00428-023-03543-4)
Supplement: Supplementary file 1 — Supplementary Table 1. Summarizing table of cell composition for each region (cells in each neighborhood / total number of cells in that neighborhood) in the 13 non-invasive IOPNs. Supplementary Table 2. Summarizing table of the number of cells and relative percentage in the two IOPNs with associated invasive adenocarcinoma. [file 428_2023_3543_MOESM1_ESM.docx]

**Supplementary Table 1**. Summarizing table of cell composition for each region (cells in each neighborhood / total number of cells in that neighborhood) in the 13 non-invasive IOPNs.

| **Cell composition for each region (cells in each neighborhood / total number of cells in that neighborhood)** | | | | | | | | | | | | | | | |
| --- | --- | --- | --- | --- | --- | --- | --- | --- | --- | --- | --- | --- | --- | --- | --- |
| **ID Case** | **1** | **2** | **3** | **4** | **5** | **6** | **7** | **8** | **9** | **10** | **11** | **12** | **13** | **Mean** | **median** |
|  | **Region 1** |  |  |  |  |  |  |  |  |  |  |  |  |  |  |
| **CD20** | 3,3 | 1,7 | 6,5 | 21,2 | 1,8 | 1,8 | 6,9 | 7,5 | 1,2 | 1,3 | 11,7 | 1,9 | 7,8 | 5,7 | 3,3 |
| **CD4** | 9,8 | 1,9 | 20 | 20,9 | 6,6 | 10 | 19,9 | 6,5 | 3,4 | 14,7 | 4 | 10,3 | 10,2 | 10,6 | 10 |
| **CD8** | 26,5 | 31,7 | 40,1 | 34,8 | 30,2 | 20,5 | 24,3 | 9,2 | 5,9 | 7,9 | 13,7 | 12 | 15 | 20,9 | 20,5 |
| **Tumor cells** | 31,7 | 37,5 | 26,6 | 44,1 | 52,4 | 31,8 | 47,8 | 4,1 | 8,9 | 6,9 | 14,1 | 8,4 | 70,2 | 29,6 | 31,7 |
|  | **Region 2** |  |  |  |  |  |  |  |  |  |  |  |  |  |  |
| **CD20** | 1,6 | 0,6 | 0,9 | 3,4 | 1,2 | 2,4 | 1,7 | 3,5 | 0,2 | 1,7 | 8,8 | 0,5 | 3,5 | 2,3 | 1,7 |
| **CD4** | 7,5 | 0,9 | 8 | 15,3 | 5 | 11 | 19 | 4,9 | 1,2 | 4,9 | 3 | 7 | 7,2 | 7,3 | 7 |
| **CD8** | 22,3 | 55,7 | 15,7 | 15,8 | 32,6 | 21,3 | 15 | 6 | 2,8 | 10,6 | 6,8 | 8,8 | 14 | 17,5 | 15 |
| **Tumor cells** | 265,8 | 317,9 | 262,2 | 283,6 | 497,9 | 327,8 | 390 | 185,1 | 291,9 | 406,8 | 258,8 | 204,5 | 280,4 | 305,6 | 283,6 |
| **Region Prevalence for each case** | | | | | | | | | | | | | | | |
|  | 1 | 2 | 3 | 4 | 5 | 6 | 7 | 8 | 9 | 10 | 11 | 12 | 13 |  |  |
| **Region number 1** | 67,06 | 77,32 | 71,53 | 86,87 | 69,06 | 59,11 | 62,18 | 65,13 | 47,48 | 15,60 | 12,63 | 96,32 | 61,74 |  |  |
| **2** | 32,94 | 22,68 | 28,47 | 13,13 | 30,94 | 40,89 | 37,82 | 34,87 | 52,53 | 84,40 | 87,37 | 3,68 | 38,26 |  |  |

Abbreviations: IOPN: intraductal oncocytic papillary neoplasm.

**Supplementary Table 2**. Summarizing table of the number of cells and relative percentage in the two IOPNs with associated invasive adenocarcinoma.

| **ID Case** | **Region** | **N. CD20** | **% CD20** | **N. CD4** | **% CD4** | **N. CD8** | **% CD8** | **N. Tumor** | **% Tumor** | **Total** |
| --- | --- | --- | --- | --- | --- | --- | --- | --- | --- | --- |
| **Case 14** | **INF** | 12487 | 2% | 24167 | 4% | 68635 | 10% | 537226 | 84% | 642515 |
|  | **NON INF** | 2589 | 3% | 9501 | 12% | 8095 | 10% | 60471 | 75% | 80656 |
|  | **WS** | 15076 | 2% | 33668 | 5% | 76730 | 11% | 597697 | 82% | 723171 |
|  |  |  |  |  |  |  |  |  |  |  |
|  | **Region** | **N. CD20** | **% CD20** | **N. CD4** | **% CD4** | **N. CD8** | **% CD8** | **N. Tumor** | **% Tumor** | **Total** |
| **Case 15** | **INF** | 76 | 0% | 118 | 1% | 1399 | 8% | 16356 | 91% | 36278 |
|  | **NON INF** | 1301 | 1% | 10783 | 8% | 21977 | 16% | 107847 | 75% | 255256 |
|  | **WS** | 1377 | 1% | 10901 | 7% | 23376 | 14% | 124203 | 78% | 291534 |

Abbreviations: IOPN: intraductal oncocytic papillary neoplasm; INF: infiltrative region; NON INF: non-infiltrative region; WS: whole-slide.
